# Territoriality drives patterns of fixed space use in Caribbean parrotfishes

**DOI:** 10.1002/ece3.9833

**Published:** 2023-02-11

**Authors:** Joshua C. Manning, Sophie J. McCoy

**Affiliations:** ^1^ Department of Earth, Ocean, and Atmospheric Sciences Florida State University Tallahassee Florida USA; ^2^ Department of Biology University of North Carolina Chapel Hill North Carolina USA

**Keywords:** home range, movement, spatial ecology, spatial overlap, stability, territory

## Abstract

Animals often occupy home ranges where they conduct daily activities. In many parrotfishes, large terminal phase (TP) males defend their diurnal (i.e., daytime) home ranges as intraspecific territories occupied by harems of initial phase (IP) females. However, we know relatively little about the exclusivity and spatial stability of these territories. We investigated diurnal home range behavior in several TPs and IPs of five common Caribbean parrotfish species on the fringing coral reefs of Bonaire, Caribbean Netherlands. We computed parrotfish home ranges to investigate differences in space use and then quantified spatial overlap of home ranges between spatially co‐occurring TPs to investigate exclusivity. We also quantified the spatial overlap of home ranges estimated from repeat tracks of a few TPs to investigate their spatial stability. We then discussed these results in the context of parrotfish social behavior. Home range sizes differed significantly among species. Spatial overlap between home ranges was lower for intraspecific than interspecific pairs of TPs. Focal TPs frequently engaged in agonistic interactions with intraspecific parrotfish and interacted longest with intraspecific TP parrotfish. This behavior suggests that exclusionary agonistic interactions may contribute to the observed patterns of low spatial overlap between home ranges. The spatial overlap of home ranges estimated from repeated tracks of several TPs of three study species was high, suggesting that home ranges were spatially stable for at least 1 month. Taken together, our results provide strong evidence that daytime parrotfish space use is constrained within fixed intraspecific territories in which territory holders have nearly exclusive access to resources. Grazing by parrotfishes maintains benthic reef substrates in early successional states that are conducive to coral larval settlement and recruitment. Behavioral constraints on parrotfish space use may drive spatial heterogeneity in grazing pressure and affect local patterns of benthic community assembly. A thorough understanding of the spatial ecology of parrotfishes is, therefore, necessary to elucidate their functional roles on coral reefs.

## Graphical abstract

We investigated the social and spatial ecology of several terminal and initial phase individuals of five common Caribbean parrotfish species. Terminal phase home ranges corresponded to fixed intraspecific territories, in which territory holders had nearly exclusive access to space. Our findings advance our knowledge of parrotfish space use and have important implications for understanding their functional roles on coral reefs.

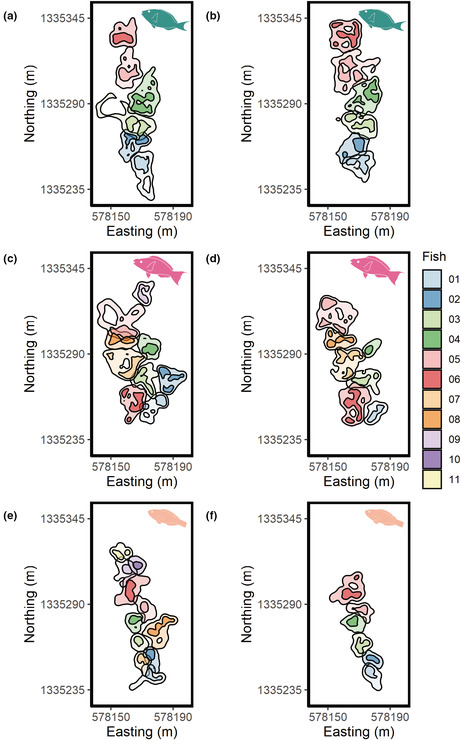

## 1 INTRODUCTION

Animals often occupy stationary, self‐constrained areas, called home ranges, that they use for daily activities (e.g., foraging and mating; Burt, 1943; Börger et al., 2008). For many taxa, home range size increases with body size, though the scaling of this relationship is affected by multiple intrinsic (e.g., locomotion, trophic status, energetic requirements), and extrinsic (e.g., resource availability) factors (Harestad & Bunnell, 1979; Haskell et al., 2002; Tamburello et al., 2015). Individuals and/or groups may maintain portions of their home ranges as territories when the benefits of priority access to mates and/or food resources exceed the costs of establishment and defense (Brown, 1964; Brown & Orians, 1970). The most basic definition of a territory is a defended area, implying that territories are maintained through agonistic interaction (Burt, 1943). However, escalated agonistic interactions (i.e., fighting) are costly and likely to be avoided, particularly when the payoffs of such interactions are small compared to the cost of escalation (Maynard Smith & Parker, 1976). Therefore, advertisement, including scent marking and visual or auditory displays, may be equally important in maintaining territory boundaries, particularly when agonistic interactions may result in death (Brashares & Arcese, 1999; Krebs et al., 1978; Lewis & Murray, 1993). Many definitions of territory also include the concept of exclusivity (i.e., intruders are not allowed access), but here we use a less restrictive definition: a fixed area of an animal's home range, maintained through social interaction, in which the owner has priority access to resources (Kaufmann, 1983).

Parrotfishes are microphages that consume epilithic and endolithic microautotrophs, primarily cyanobacteria (Cissell et al., 2019; Clements et al., 2016; Manning & McCoy, 2022; Nicholson & Clements, 2020, 2021), but some species (i.e., *Sparisoma* spp.) also consume fleshy macroalgae (Adam et al., 2015; Dell et al., 2020; Manning & McCoy, 2022). Parrotfish grazing is considered important for maintaining cropped, early successional substrates that are conducive to coral larval settlement and recruitment, thereby enhancing reef recovery and resilience (Bonaldo et al., 2014; Mumby et al., 2007). However, parrotfishes strongly partition trophic resources (Adam et al., 2015; Nicholson & Clements, 2020, 2021), and foraging rates, food intake, and the likelihood of producing grazing scars (i.e., bare space) vary among species and as a function of body size (Adam et al., 2015, 2018; Bruggemann, Begeman, et al., 1994; Bruggemann, Kuyper, & Breeman, 1994). As such, their effects on benthic communities are likely to be species and size‐specific.

Parrotfishes are protogynous hermaphrodites, and many species have two distinct color phases: (1) initial phase (IP), drab‐colored females and primary males (in diandric species) and (2) terminal phase (TP), colorful secondary males (Robertson & Warner, 1978). They transition from IP to TP, usually within a certain size class; as such, TPs are generally larger than IPs of the same age (Choat et al., 1996, 2003; Robertson & Warner, 1978). In many parrotfishes, TPs defend diurnal (i.e., daytime) foraging areas containing harems of IPs (typically 1–7) from other intraspecific TPs (Buckman & Ogden, 1973; Mumby & Wabnitz, 2002; van Rooij et al., 1996). This territorial behavior is costly for parrotfishes (i.e., reduced body condition), but may increase access to mates, mating sites, and/or food resources (Bruggemann, van Oppen, & Breeman, 1994; van Rooij et al., 1995, 1996). Parrotfishes often show strong site fidelity to their diurnal home ranges/territories (hereafter, just home ranges), returning daily after nocturnal migrations to and from sleeping sites (Ogden & Buckman, 1973; Pickholtz et al., 2022; van Rooij et al., 1996). However, we still know relatively little about the exclusivity and spatial stability of parrotfish home ranges, despite the potential importance of herbivore space use in driving patterns of benthic community assembly (Sandin & McNamara, 2012).

Here, we ask: (1) how does home range size differ within and among Caribbean parrotfish species, and (2) how exclusive and spatially stable are these home ranges? To answer these questions, we conducted concurrent GPS tracking and behavioral observations of territorial TPs and IPs of five common parrotfish species across five fringing coral reefs in Bonaire, Caribbean Netherlands. Specifically, we tested the predictions that (1) the home range sizes of territorial parrotfishes would differ by species and ontogenetic phase (TP and IP), (2) the spatial overlap among home ranges of intraspecific pairs of TPs would be low relative to overlap between interspecific pairs, and (3) these home ranges would remain fixed for extended periods of time. The third prediction assumed temporal stability of resources, a reasonable assumption given that there were no disturbances (e.g., coral bleaching) during the study period. Finally, we discuss data on social interactions among parrotfishes to provide context for our results.

## 2 METHODS

### 2.1 Study species and sites

We conducted our research on *Scarus iseri*, *Scarus taeniopterus*, *Scarus vetula*, *Sparisoma aurofrenatum*, and *Sparisoma viride* at five fringing coral reefs along the leeward coast of Bonaire during January (Winter) and May–July (Summer) 2019: Angel City (12.10305°, −68.28852°), Aquarius (12.09824°, −68.28624°), Bachelor's Beach (12.12605°, −68.28819°), Invisibles (12.07805°, −68.28175°), and The Lake (12.10618°, −68.29079°). The fringing coral reefs of Bonaire, Caribbean Netherlands have remained resilient despite multiple disturbances, and boast higher coral cover than most Caribbean coral reefs (Perry et al., 2013; Steneck et al., 2019). The abundance and biomass of different fish groups, including parrotfishes, are also much higher on Bonaire's coral reefs compared to more heavily fished reefs in the Eastern Caribbean (Hawkins & Roberts, 2003, 2004; Steneck et al., 2019). The benthic composition across our study sites was similar, with relatively high coral cover (~20%) and low macroalgal cover (<3%; Manning & McCoy, 2022). Additionally, our five focal parrotfish species comprised more than 96% of the parrotfish biomass at our study sites (Manning & McCoy, 2022).

### 2.2 Parrotfish tracking

We conducted concurrent GPS tracking and behavioral observations of TP and IP parrotfishes between 1000 and 1600 h, peak foraging times for parrotfishes (Bruggemann, Begeman, et al., 1994; Bruggemann, Kuyper, & Breeman, 1994). We identified focal parrotfish (TP or IP) haphazardly at ~10 m depth on SCUBA at each site. Each fish was then allowed to acclimate to diver presence for approximately 1–2 min, during which time we visually estimated standard length (to the nearest cm) by measuring the distance between reference objects passed by the fish using a collapsible meter stick. We then estimated body mass (g) from standard length using published length‐weight relationships (Bohnsack & Harper, 1988; Appendix A: Table A1). We followed focal fish from ~2 m, and recorded their behavior in high resolution (4K) using a GoPro Hero 4 Silver (GoPro, Inc) attached to a ‘selfie‐stick’.

Focal parrotfishes were tracked at the surface by a snorkeler carrying a handheld GPS receiver (Garmin GPSMap 78sc, United States of America) for 13.56 ± 0.19 mins (mean ± SE, *n* = 215 total tracks). The GPS receiver was set to record data as often as possible, resulting in a mean (±SE) relocation interval of 12.32 ± 0.21 s (mean ± SE, *n* = 215 total tracks). We ensured that we did not track the same individuals unintentionally by progressively moving north along the reef, using reference structures, until we identified another unique individual to observe. A few times, we unintentionally conducted repeat tracks of previously tracked individuals (on the same day or within a few days; confirmed as described below). In such cases, unintentional repeat tracks were excluded from our analyses. Because our interest was in the home range behavior of territorial fishes, we excluded non‐territorial, transient TP fishes from our analyses. Transient, non‐territorial TPs were not site attached and were frequently chased along the reef by territory holders.

**FIGURE A1:**
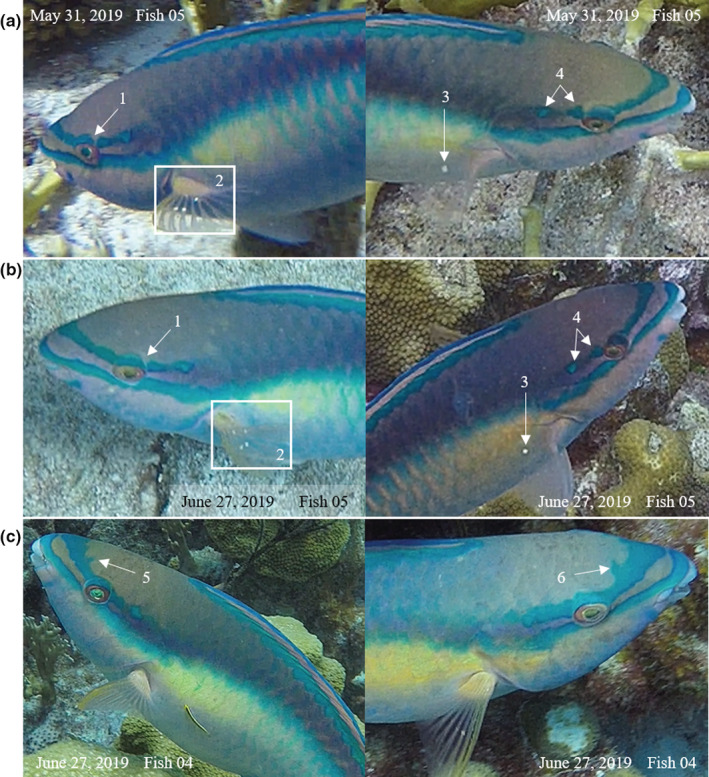
Examples of identifying markings used to distinguish TP *Sp. viride* during repeated tracking. Panel (a) shows photos of an individual (05 in Figure 1) tracked on May 24, 2019 at Invisibles. Panel (b) shows photos of the same individual tracked 28 days later on June 21, 2019. Key identifying markings of this individual are highlighted by shared numbers in these panels [1, 2, 3]. For example, [2] shows a unique gap in the reddish band running from the fish's mouth. Panel (c) shows photos of a neighboring individual (06 in Figure 1) and key identifying markings that differ from those of its neighbor. Note the thicker band running behind the left eye [4], the thin bands running parallel to the gills [5], and wings on the yellow scales of the caudle peduncle [6].

**FIGURE A2:**
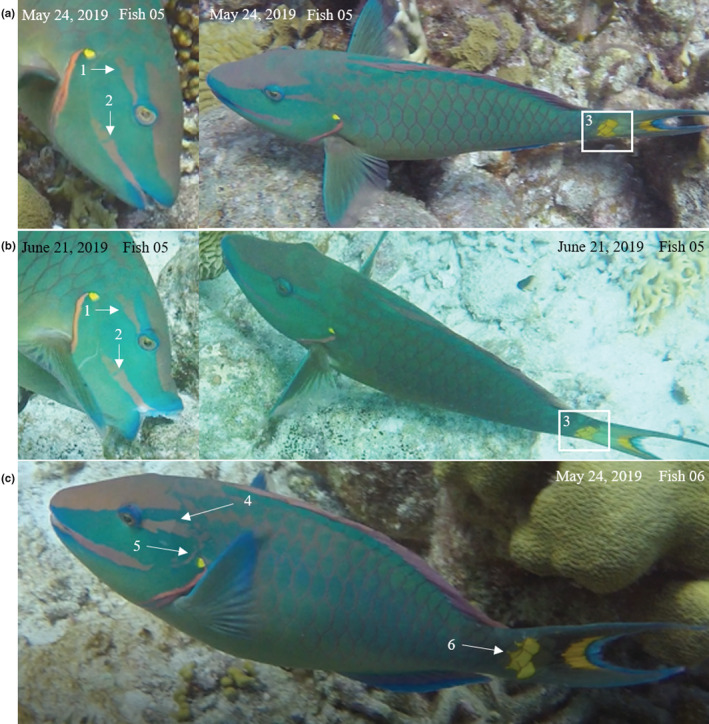
Examples of identifying markings used to distinguish TP *Sc. vetula* during repeated tracking. Panel (a) shows photos of a fish (07 in Figure 1) tracked on May 31, 2019 at Invisibles. Panel (b) shows photos of the same fish tracked 25 days later on June 25, 2019. Key identifying markings of this individual are highlighted by shared numbers in these panels [1, 2, 3, 4]. For example, [3] shows three unique blue spots behind the fish's mouth. Panel (c) shows photos of a neighboring fish (08 in Figure 1) and key identifying markings that differ from those of its neighbor. Note the small spot at the top of the head [5], the blue dot on the band above the right eye [6], and no blue dot on the band above the left eye [7].

**FIGURE A3:**
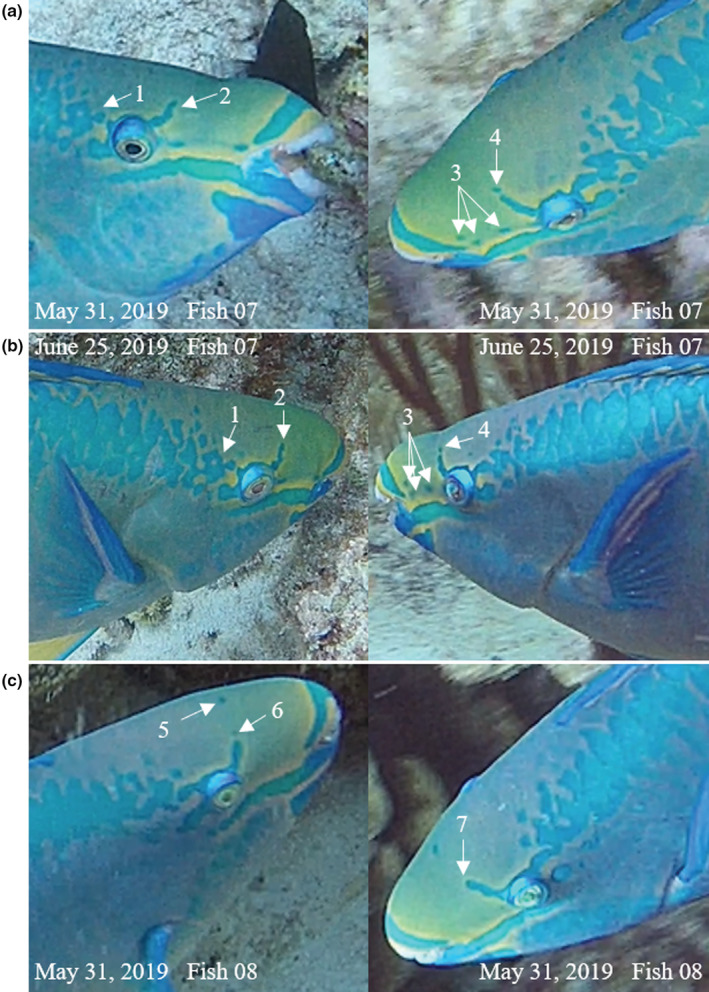
Examples of identifying markings used to distinguish TP *Sc. taeniopterus* during repeated tracking. Panel (a) shows photos of a fish (05 in Figure 1) tracked on May 31, 2019 at Invisibles. Panel (b) shows photos of the same fish tracked 27 days later on June 27, 2019. Key identifying markings of this individual are highlighted by shared numbers in these panels [1, 2, 3, 4]. For example, [2] shows three unique white spots on the fish's pectoral fin. Panel (c) shows photos of a neighboring fish (04 in Figure 1) and key identifying markings that differ from those of its neighbor. Note the wider blue band running across the fish's head, which also has longer protrusions than in the other individual [5, 6].

We preliminarily tracked several territorial TP (hereafter, just TP) *Sp. viride* at two sites in Winter 2019. Then, during Summer 2019, we tracked several TP *Sc. taeniopterus*, *Sc. vetula*, and *Sp. viride* at all five sites, and TP *Sc. iseri* and *Sp. aurofrenatum* at two sites. To investigate differences in space use among ontogenetic phases, we also tracked IP *Sc. taeniopterus*, *Sc. vetula*, and *Sp. viride* at two sites during Summer 2019. Finally, to quantify the short‐term spatial stability of parrotfish home ranges (described below), we conducted planned repeat tracks of several TP *Sc. taeniopterus*, *Sc. vetula*, and *Sp. viride* at two sites during Summer 2019. Repeat tracks were obtained by tracking TPs along the same portions of the reef where they had been tracked ~1 month prior. Individual parrotfish are identifiable by unique color patterns and markings on their bodies (Dubin, 1981; van Rooij et al., 1996). We compared the color patterns and markings of each fish using stills taken from our video‐recorded behavioral observations to confirm that initial and repeat tracks were of the same fish and not different fish occupying the same areas (Figures A1, A2, A3). We also obtained unplanned, repeat tracks of 4 TP *Sp. viride* at Angel City and Bachelor's Beach (*n* = 2 per site) during Summer 2019 that was initially tracked in January 2019. Home ranges of the 4 fish were in the same locations in both tracking periods, and visual observation of color patterns and markings from video stills confirmed that they were the same fish. A full breakdown of our sampling effort is reported in the Appendix A (Table A2).

### 2.3 Home range estimation

**FIGURE A4:**
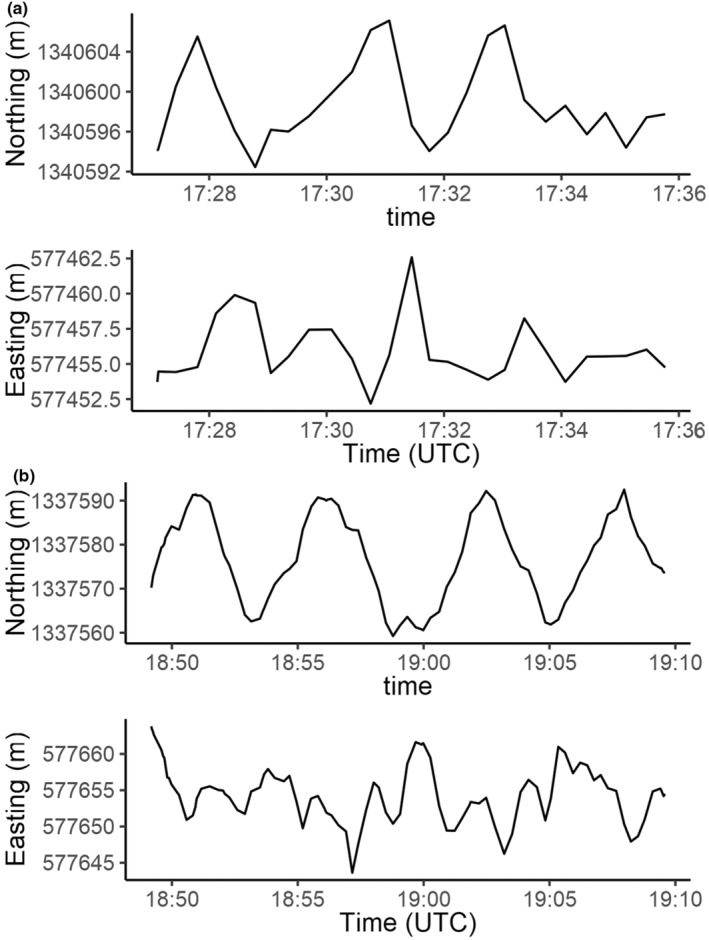
Time (UTC) series plots demonstrating the locational stationarity [Northing (m) and Easting (m); UTM 19N projection, EPSG: 32619] of a TP *Sc. taeniopterus* (a) tracked for 8.65 min at Bachelor's Beach and a TP *Sp. viride* (b) tracked for 20.40 min at Aquarius in Bonaire, Caribbean Netherlands during Summer 2019. Note that the mean and variance are relatively stable across both time series, suggesting stationary movements despite variation in tracking time.

Visual analyses of stationarity confirmed that our GPS tracks were sufficiently long to capture home range behavior (Figure A4; Benhamou, 2014). We used movement‐based kernel density estimation (MKDE) to estimate utilization distributions from our tracks of individual parrotfishes (sensu Benhamou, 2011), and define home range and core use areas as the areas underneath the 95% and 50% cumulative isopleths of each utilization distribution, respectively. Though we used MKDE estimates of home ranges for analyses of space use in our study, we also present home range sizes estimated using traditional location‐based kernel density estimation (ad hoc bandwidth selection) and minimum convex polygons to facilitate comparisons of home range area with other studies (Table A3). All home ranges and core use areas were computed in the adehabitatHR package in R (Calenge, 2006; R Core Team, 2020).

### 2.4 Home range exclusivity and stability

To investigate the exclusivity of parrotfish home ranges, we quantified spatial overlap between home ranges of spatially co‐occurring TP parrotfishes tracked during Summer 2019. This measure of spatial overlap estimates shared space to use between neighboring fish that are sharing at least some space on the reef. We used only home ranges estimated from the first GPS track of TPs for which we had replicated GPS tracks. Spatial overlap was estimated using Bhattacharyya's Affinity (BA), a function of the product of two utilization distributions that range from 0 (no overlap) to 1 (perfect overlap) and is a strong metric of joint space use between animals, particularly when comparing utilization distributions estimated for the same animal at different times (Fieberg & Kochanny, 2005). As such, we also used BA to quantify the spatial overlap of home ranges estimated from repeat tracks of TP *Sc. taeniopterus*, *Sc. vetula*, and *Sp. viride* to determine the spatial stability of those home ranges. To distinguish spatial overlap between different spatially co‐occurring individuals and spatial overlap of home ranges estimated from repeat tracks of the same individuals, we use the terms spatial overlap and temporal overlap, respectively. Spatial and temporal overlaps of MKDE home ranges were computed in the adehabitatHR package in R (Calenge, 2006).

### 2.5 Occupancy change

**FIGURE A5:**
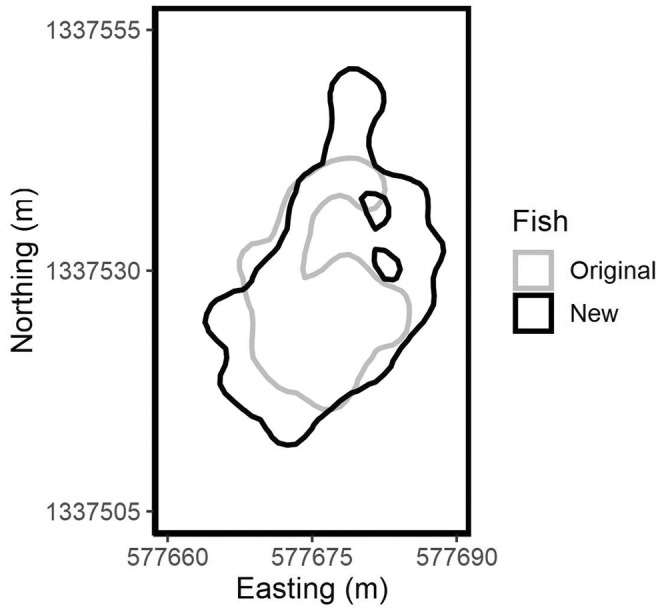
A map showing the home ranges of the original (gray) and new occupant (black) of the TP *Sc. vetula* territory at Aquarius where we observed an occupancy change. Mapped using the UTM 19N (EPSG: 32619) projection.

During our attempt to repeatedly track parrotfishes at Aquarius, we observed an occupancy change in a TP *Sc. vetula* home range. We analyzed this occupancy change as a separate case study (Figure A5). To investigate how change in occupancy affected space use, we quantified spatial overlap between the home range of the new occupant and the home range of the old occupant. We also quantified spatial overlap between the home range of the new occupant and the home ranges of spatially co‐occurring intraspecific and interspecific fishes from the initial tracking (Table A4).

### 2.6 Social behavior

We quantified the social behavior of the TP parrotfishes tracked in Summer 2019 (*n* = 128) by analyzing the video recordings of the initial tracks for each fish (when repeat tracks existed) in the behavioral software BORIS (v. 7.9.8; Friard & Gamba, 2016). For each fish, we quantified the number of agonistic interactions they had with other parrotfishes, the identity (i.e., ontogenetic phase and species) of interacting parrotfishes, and interaction durations (s). In some cases, we were unable to determine the identity of the interacting parrotfish and classified such interactions as ‘unknown’. Agonistic interactions between parrotfishes consisted of displays (i.e., extension of dorsal and pelvic fins) and more aggressive (and typically longer) chases that are characteristic of territorial behavior/aggression in parrotfishes (e.g., Buckman & Ogden, 1973; Mumby & Wabnitz, 2002).

### 2.7 Statistical analyses

All statistical models were fit in the ‘glmmTMB’ package (v. 1.0.2.1; Brooks et al., 2017) in R (v. 4.0.2 R Core Team, 2020). To investigate differences in home range sizes, we used generalized linear mixed models fit gamma distributions with log‐link functions. We used only the home ranges estimated from initial tracks of parrotfishes conducted in Summer 2019 for these analyses. We first fit a model for the three species for which we had IP observations to provide a comprehensive investigation of the importance of the ontogenetic phase, as well as species and body size. This model included species, ontogenetic phase, log body mass, and their interactions as fixed effects. We then fit another model for all five species that included species, log body mass, and their interaction as fixed effects. Both models included site as a random effect and log of track duration as an offset to account for the potential influence of unequal track length on home range size. We conducted model selection for both global models using corrected Akaike's Information Criterion (AICc). When multiple best‐fit models were obtained (i.e., ΔAICc <2), the most parsimonious model was used for inference. Graphical analysis of residuals confirmed that model assumptions were met for all models.

To investigate differences in spatial and temporal overlap, we used generalized linear models fit beta distributions with logit link functions. The beta distribution is defined by two parameters, mean (*μ*) and precision (*θ*), the latter of which can be modeled as a constant or allowed to vary (i.e., fixed or variable dispersion; Bayer & Cribari‐Neto, 2017; Ferrari & Cribari‐Neto, 2004). Incorrectly modeling dispersion as fixed can result in increased variance in the estimation of model coefficients (Bayer & Cribari‐Neto, 2017). Therefore, we conducted model selection (AICc) for each beta regression in two steps (sensu Bayer & Cribari‐Neto, 2017): (1) we conducted model selection of nested fixed dispersion models (i.e., *θ* fixed) to determine which predictors to include in the mean equation of the model; (2) we conducted model selection to determine whether or not variable dispersion models were necessary, and to determine which explanatory variables (determined in step 1) to include in the precision equation of the model. As above, when two or more best‐fit models were found (ΔAICc <2), the most parsimonious model was selected for inference. For our analyses of spatial overlap (home range exclusivity), fixed effects in the global fixed dispersion model included site, pairwise identity of the interactors (15 pairwise species combinations; e.g., Species A–Species B), and the time interval (days) between the tracks used to estimate the home ranges for each fish. We excluded spatial overlap estimates involving the individual that took ownership of one of the initial *Sc. vetula* home ranges at Aquarius from these analyses. For our analyses of temporal overlap (home range stability), fixed effects in the global model included site, species (*Sc. taeniopterus*, *Sc. vetula*, *Sp. viride*), and the time interval (days) between the initial and repeat tracks used to estimate the home ranges for each fish.

To provide social context for patterns of parrotfish space use, we quantified the frequency and duration of social interactions between parrotfishes (*n* = 128 initial observations of focal TPs in Summer 2019). We investigated differences in the frequency of interactions between focal TPs and other parrotfishes using a generalized linear mixed model fit to a negative binomial distribution with the log of observation time included as an offset and the site as a random effect. We defined observation time (13.49 ± 0.23 min) as the total time of the video recorded observation (13.59 ± 0.23 min) minus any time in which the fish was not visible in the video recording (0.10 ± 0.02 min; mean ± SE for *n* = 128 TP observations). We limited our analyses to interactions for which we had positively identified the parrotfish with which the focal TP fish was interacting. We also excluded one interaction that involved a focal TP *Sp. aurofrenatum* interacting with multiple individuals of two species, *Sparisoma chrysopterum* and *Sparisoma rubripinne*, at the same time because of the uncertainty of the specific target of agonistic behavior. We recorded parrotfish interactions (identity of interactor known) in only 114 of the 128 focal TP observations and limited our analyses to these fishes. We then analyzed differences in the duration of interactions between focal TPs and other parrotfishes using a generalized linear mixed model fit to a gamma distribution with a log‐link function and focal fish identity nested within the site as a random effect. Our full models included focal species, interactor type (e.g., IP—intraspecific), and the interaction between focal species and interactor type as fixed effects. We conducted model selection for both global models using AICc. When multiple best‐fit models were obtained (i.e., ΔAICc <2), the most parsimonious model was used for inference. Graphical analysis of residuals confirmed that model assumptions were met for all models.

## 3 RESULTS

### 3.1 Tracking summary

In total, we obtained GPS tracks of 175 unique individuals of five species, *Sc. taeniopterus*, *Sc. vetula*, *Sp. viride*, *Sc. iseri*, and *Sp. aurofrenatum*; and repeat GPS tracks of 40 individuals of three species, *Sc. taeniopterus*, *Sc. vetula*, and *Sp. viride* (Table A5). Movement appeared to be recursive as individuals repeatedly foraged in the same locations throughout the tracks. Additionally, a visual assessment confirmed that their movements were largely stationary (Figure A4). As such, we are confident that these tracks adequately capture diurnal home range movements.

### 3.2 Home range size

**FIGURE 1:**
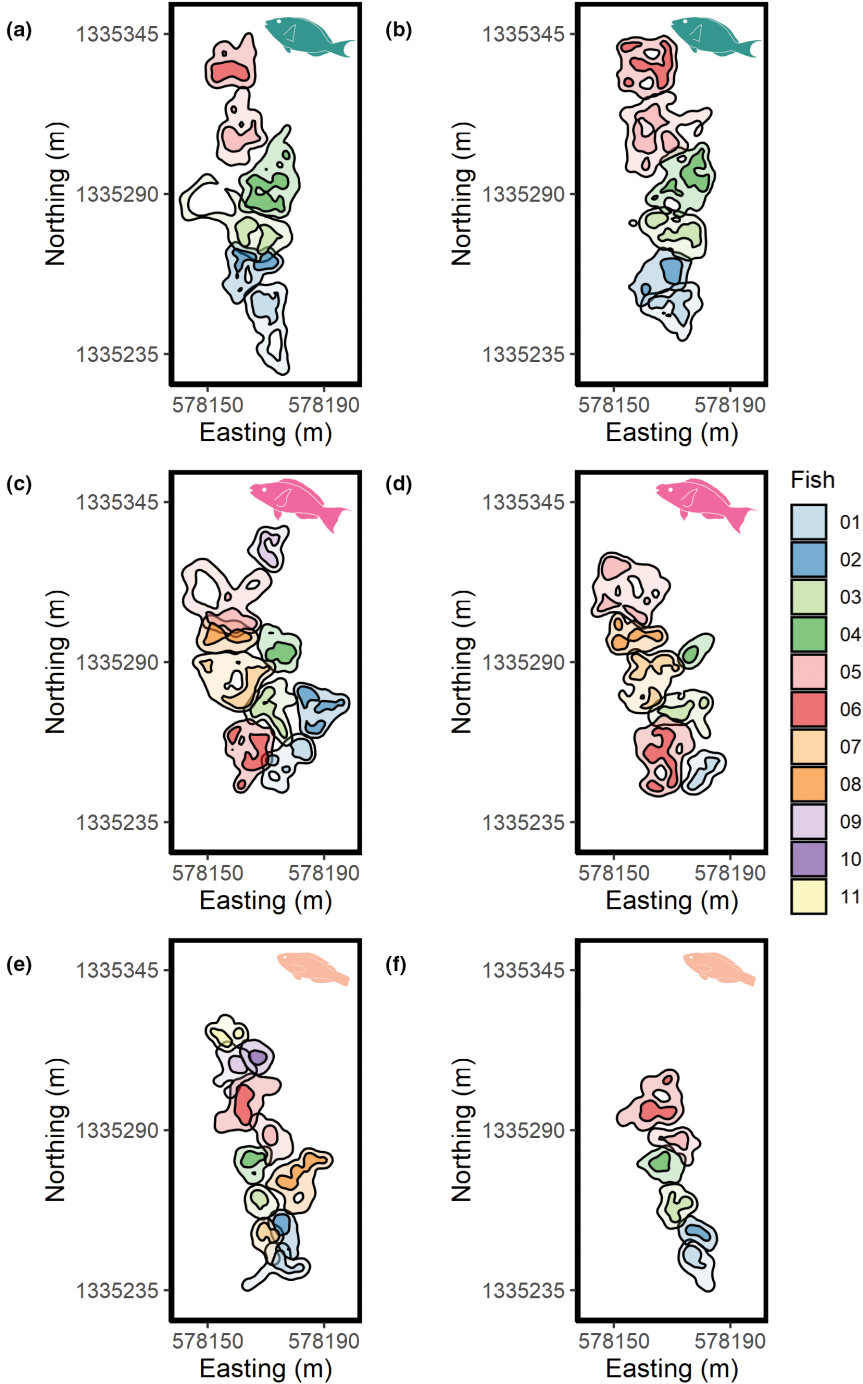
Maps of home ranges and core use areas (shaded darker) estimated from initial (left) and repeat (right) tracks of TP *Sp. viride* (a, b), *Sc. vetula* (c, d), and *Sc. taeniopterus* (e, f) at Invisibles fringing reef during Summer 2019. Each individual of a species is a unique color and colors are consistent between initial and repeat tracks. All maps are presented on the same scale using the same projection (UTM 19N, EPSG: 32619).

**FIGURE 2:**
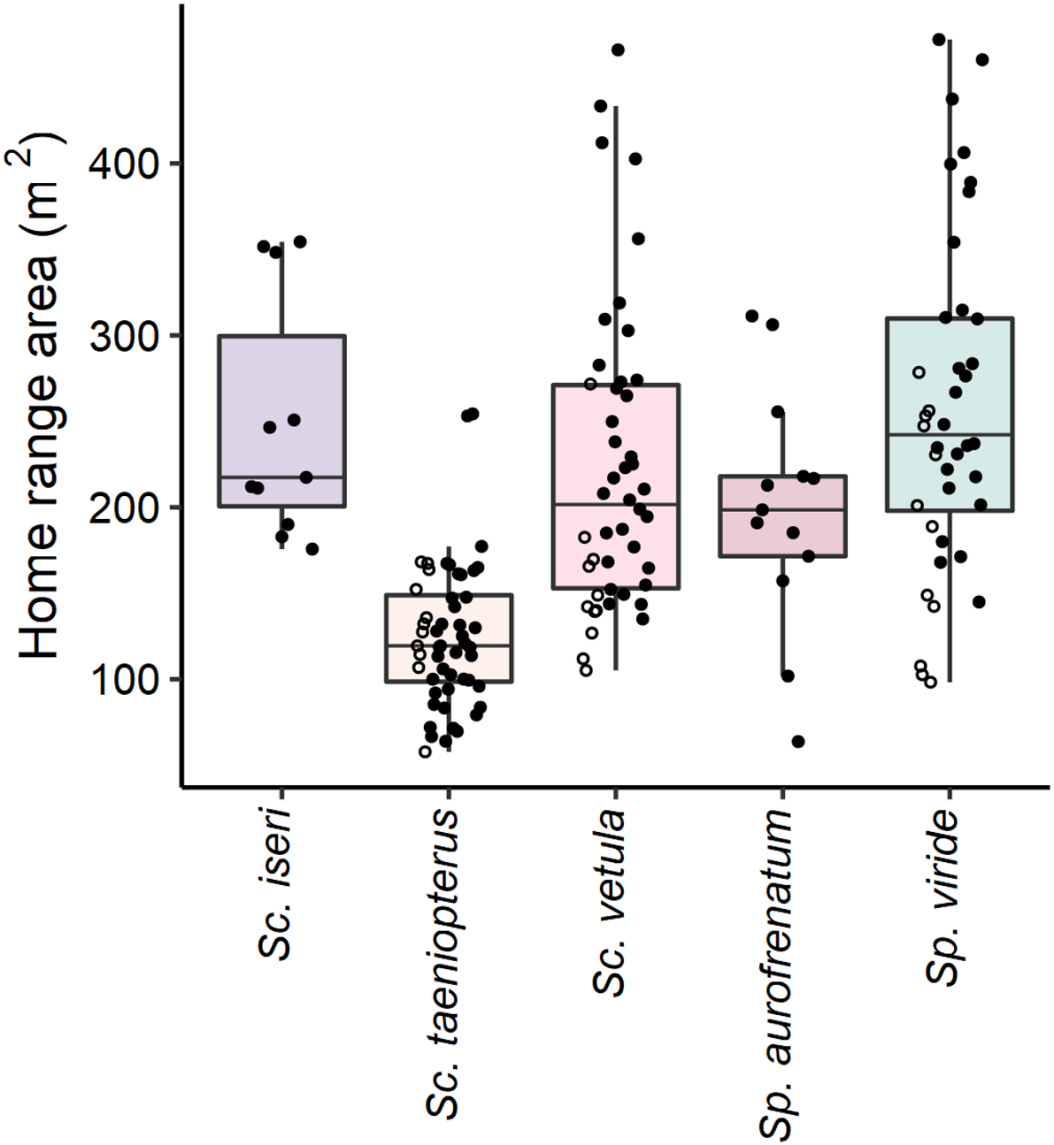
Boxplots of home range areas (m^2^) for *Sc. iseri*, *Sc. taeniopterus*, *Sc. vetula*, *Sp. aurofrenatum*, and *Sp. viride* (color coded). The points represent the home range areas of individual parrotfishes: open circles are IP fish and closed circles are TP fish.

The best‐fit model for the home range area including the three species for which we had IP observations contained only species as a predictor, though a model including the additive effects of species and ontogenetic phase was similarly well fit (ΔAICc = 1.84). The best‐fit model for the home range area including all five species also contained only species as a predictor, though a model including the additive effect of species and log body mass was similarly well fit (ΔAICc = 1.75). Because the best fit and most parsimonious model in both cases included only species, we will only discuss the results of the model that included data for all five species and species as the only predictor. There were significant differences in home range areas among species (Type III Wald's *χ* ^2^ = 34.49, *df* = 4, *p* < .001; Table A6). *Scarus taeniopterus* had significantly smaller home ranges than *Sc. vetula* and *Sp. viride*, and the home ranges of *Sc. iseri* and *Sp. aurofrenatum* were intermediately sized (Figures 1 and 2).

### 3.3 Home range exclusivity

**FIGURE 3:**
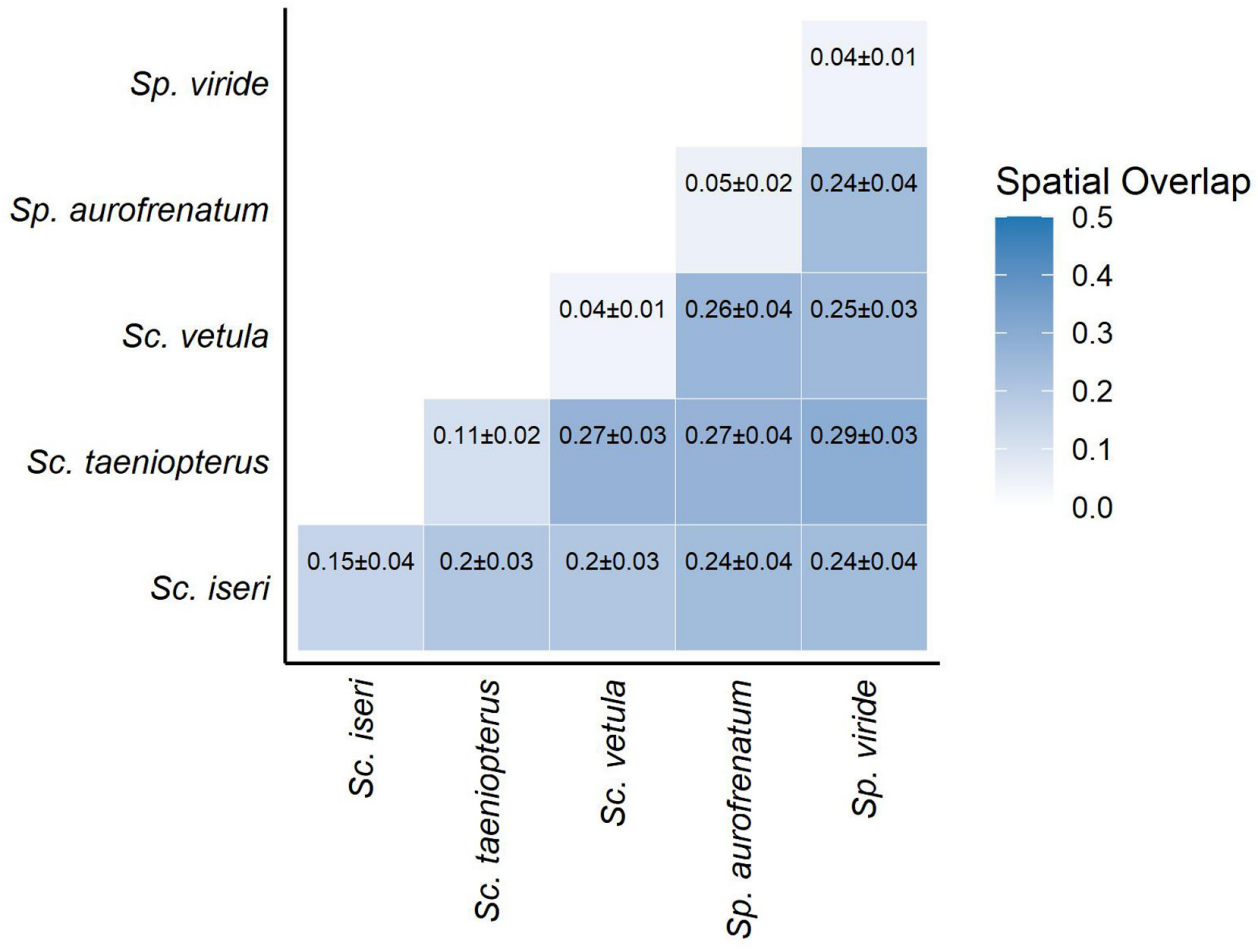
A heatmap of mean (±SE) spatial overlaps (BA) for spatially co‐occurring pairs of TP parrotfishes. Light colors indicate low spatial overlap and dark colors indicate greater spatial overlap.

**FIGURE A6:**
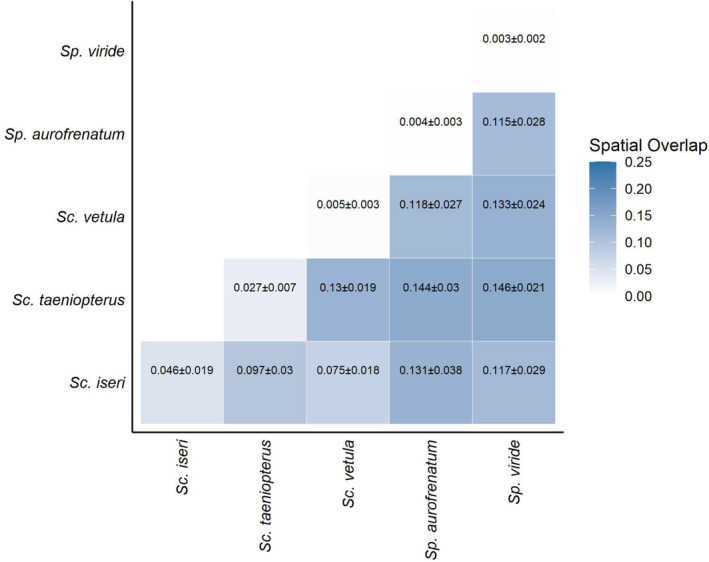
A heatmap of mean (±SE) spatial overlaps (UDOI) for spatially co‐occurring pairs of TP parrotfishes. Light colors indicate low spatial overlap and dark colors indicate greater spatial overlap.

The best fit and most parsimonious fixed dispersion model for spatial overlap included only pairwise interaction in the mean component of the model, though a model including pairwise interaction and number of days between tracks as predictors in the mean component was similarly well fit (ΔAICc = 1.81). The variable dispersion model including pairwise interaction in both the mean and precision components of the model was a much better fit than a fixed dispersion model including pairwise interaction in only the mean component of the model (ΔAICc >10). Generally, the home ranges of intraspecific pairs of TP parrotfish overlapped very little (0.09 ± 0.01, mean ± SE spatial overlap, *n* = 98) relative to the home ranges of interspecific pairs of TP parrotfishes (0.25 ± 0.01, *n* = 423). However, this general pattern of spatial overlap of home ranges was dependent upon the identity of the interacting pairs (Type III Wald's *χ* ^2^ = 137.39, *df* = 14, *p* < .001; Table A7). Specifically, there was no significant difference in the extent of spatial overlap between home ranges of intraspecific pairs of TP *Sc. iseri* and home ranges of interspecific pairs that included TP *Sc. iseri*, regardless of species (Figure 3). The spatial overlap between intraspecific home ranges was particularly low for TP *Sc. vetula*, *Sp. aurofrenatum*, and *Sp. viride* (0.04 ± 0.01, 0.05 ± 0.02, and 0.04 ± 0.01, respectively; mean ± SE; Figure 1), much lower than the spatial overlap between home ranges of interspecific pairs including these species (Figure 3). The use of the utilization distribution overlap index (UDOI; Fieberg & Kochanny, 2005) rather than BA does not affect these interpretations (Figure A6).

### 3.4 Home range stability

The best‐fit fixed dispersion model of temporal overlap included the site as a predictor in the mean component of the model. A fixed dispersion model including site and number of days between repeated tracks fit similarly well (ΔAICc = 0.93). However, the intercept‐only model was most parsimonious and also fit well (ΔAICc = 0.26). Home ranges were relatively stable throughout the study period. Home ranges estimated from repeat tracks of TP *Sc. taeniopterus*, *Sc. vetula*, and *Sp. viride* overlapped substantially with the home ranges estimated from their initial tracks conducted 18–20 days prior [short‐term temporal overlap: 0.59 ± 0.04 (14), 0.68 ± 0.05 (11), and 0.62 ± 0.04 (11); mean ± SE (*n*), respectively]. Additionally, four TP *Sp. viride* tracked during January 2019 at two of our sites were tracked again 121–139 days later during Summer 2019. The long‐term temporal overlap of the home ranges estimated from these repeated tracks of TP *Sp. viride* was also high (0.64 ± 0.07, mean ± SE, *n* = 4) and equivalent to the short‐term temporal overlap of TP *Sp. viride* home ranges.

### 3.5 Occupancy change

During our effort to conduct repeat tracks of TP *Sc. vetula* at one site, we observed an occupancy change in one home range. The new occupant's home range overlapped substantially with the home range of the original occupant (spatial overlap = 0.68; Figure A5). The new occupant's home range overlapped very little with one neighboring intraspecific home range (spatial overlap = 0.001), reflecting the low home range overlap of other spatially co‐occurring TP *Sc. vetula*. Likewise, the spatial overlap between the new occupant's home range and the home ranges of spatially co‐occurring interspecific parrotfishes at Aquarius (0.25 ± 0.06, mean ± SE, *n* = 15) was not different from the home range overlap of spatially co‐occurring interspecific pairs across all pairwise species combinations.

### 3.6 Social behavior

In total, we observed 42, 142, 106, 28, and 83 agonistic interactions between focal TP *Sc. iseri*, *Sc. taeniopterus*, *Sc. vetula*, *Sp. aurofrenatum*, and *Sp. viride* (respectively) and other parrotfishes (positively identified). We observed another 6, 14, 17, 5, and 15 instances when focal TP *Sc. iseri*, *Sc. taeniopterus*, *Sc. vetula*, *Sp. aurofrenatum*, and *Sp. viride* (respectively) were behaving in a manner that indicated that they were interacting agonistically with another parrotfish (i.e., similar chase/display behavior), but we were unable to identify the interactor confidently from our videos. We focused our analyses only on agonistic interactions in which the interactor identity was known. In one TP *Sp. viride* home range at Angel City, the focal appeared to share its home range with two other TP *Sp. viride*. We did not observe any apparent agonistic interactions between these three TPs, but the focal TP was observed interacting with other TP *Sp. viride*, suggesting defense of this shared home range.

**FIGURE 4:**
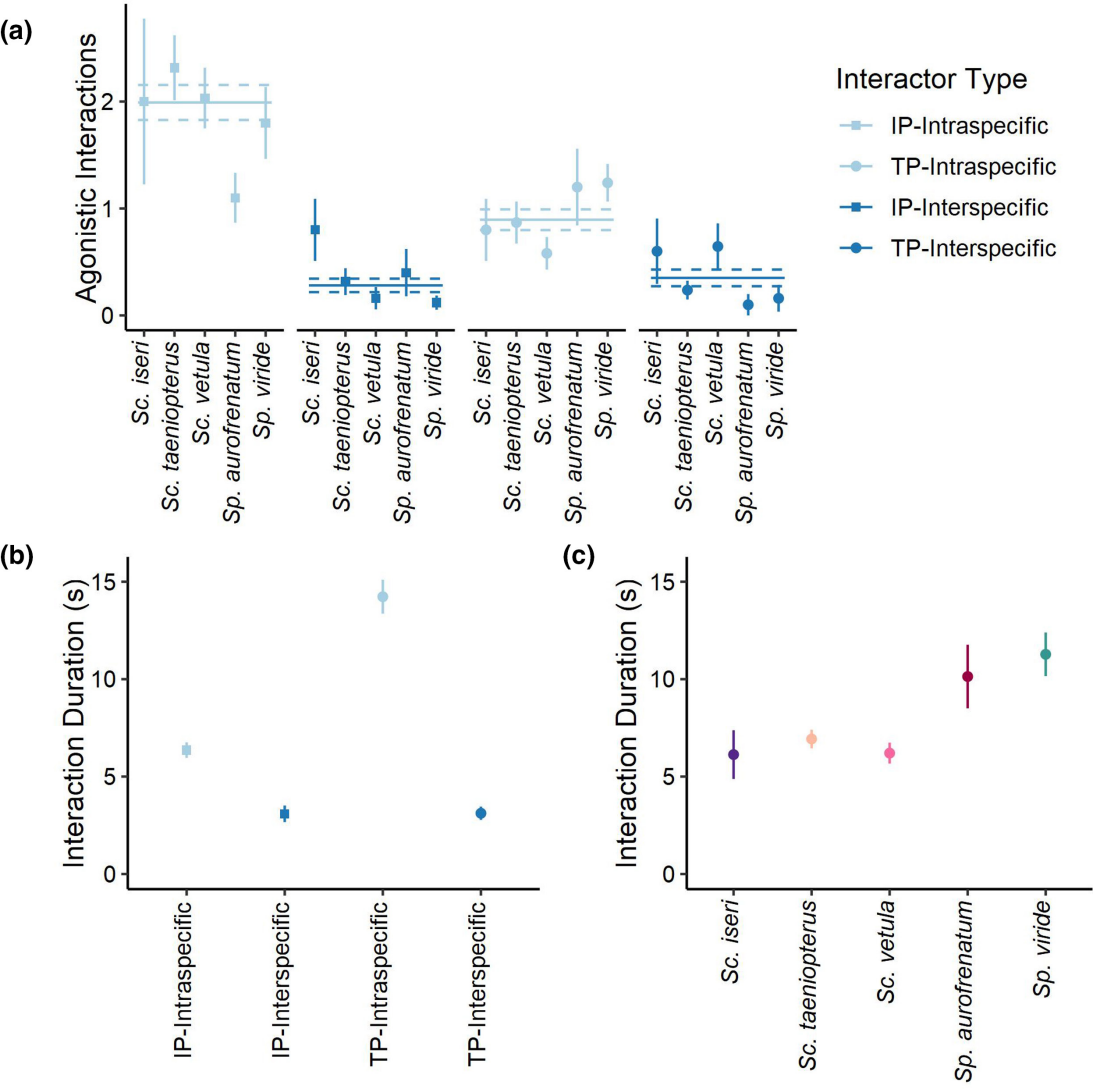
(a) Mean (±SE) number of agonistic interactions between focal TP *Sc. iseri*, *Sc. taeniopterus*, *Sc. vetula*, *Sp. aurofrenatum*, and *Sp. viride* and either TP or IP intra‐ and interspecific parrotfishes. The mean (±SE) number of agonistic interactions with TP or IP intra‐ and interspecific parrotfishes across all species are shown as solid (mean) and dashed (SE) lines. (b) Mean (±SE) duration of agonistic interactions between focal TP parrotfishes and TP or IP intra‐ and interspecific parrotfishes. (c) Mean (±SE) duration of agonistic interactions for focal TP *Sc. iseri*, *Sc. taeniopterus*, *Sc. vetula*, *Sp. aurofrenatum*, and *Sp. viride*.

The best‐fit model for our analysis interaction frequency included focal species, interactor type (e.g., TP—intraspecific), and their interaction. We found a significant interactive effect of focal species and interactor type on the frequency of agonistic interactions between parrotfishes (Type III Wald's *χ* ^2^ = 30.50, *df* = 12, *p* = .002; Table A8), suggesting that the number of interactions with different interactor types varied by species (Figure 4a). Focal TP *Sc. taeniopterus* and *Sc. vetula* interacted most frequently with intraspecific IP parrotfish, and focal TP *Sp. viride* interacted most frequently with intraspecific IP and TP parrotfish. Focal TP *Sc. iseri* and *Sp. aurofrenatum* interacted equally often with intra‐ and interspecific parrotfishes.

The best‐fit model for our analysis of the duration of agonistic interactions between parrotfishes included only focal species and interactor type (e.g., TP – intraspecific) as fixed effects. The duration of agonistic interactions between focal TPs and other parrotfishes varied by species (Type III Wald's *χ* ^2^ = 12.86, *df* = 4, *p* = .01) and interactor type (Type III Wald's *χ* ^2^ = 165.08, *df* = 3, *p* < .001; Table A9). Agonistic interactions between focal TPs and other parrotfishes were generally longer for the two *Sparisoma* spp. than for the *Scarus* spp. (Figure 4c). Additionally, agonistic interactions between focal TPs and intraspecific parrotfish were longer than those between focal TPs and interspecific parrotfishes and focal TPs interacted longest with TP intraspecific parrotfish (Figure 4b).

## 4 DISCUSSION

We leveraged concurrently collected space use and behavioral data to investigate home range behavior and territoriality in Caribbean parrotfishes. We found that parrotfish space use varied significantly among species. Specifically, we found that *Sc. taeniopterus* had smaller home ranges (i.e., territories) than *Sc. vetula* and *Sp. viride*, while *Scarus iseri* and *Sp. aurofrenatum* had intermediately sized territories. Additionally, there was some support for the inclusion of ontogenetic phase (three species model) and log of body mass (five species model) in our models of home range size. Welsh et al. (2013) found that the home ranges of parrotfishes rapidly increased in area as a function of body size until fish reached sexual maturity at 10–15 cm total length, after which the rate of increase was substantially reduced. The mean body sizes of the fishes we tracked were greater than this threshold (Table A1), which may explain why there was no stronger support for the inclusion of these predictors in our models. Regardless, smaller IPs tended to have smaller home ranges than the larger TPs (Figure 2). This was particularly evident in *Sc. vetula* and *Sp. viride*, for which the difference in body size was greatest between the tracked IPs and TPs (Table A1).

Intraspecific TPs interacted longest with one another and the spatial overlap between their intraspecific TP home ranges was also the lowest, particularly for *Sc. vetula*, *Sp. aurofrenatum*, and *Sp. viride*. Taken together this may mean that the length of interaction is indicative of aggressive exclusion or territoriality. Low spatial overlap between home ranges of intraspecific pairs of TPs suggests that territory holders have priority access to resources within their home ranges. Focal TPs most frequently interacted agonistically with intraspecific fish, consistent with prior studies of territoriality in parrotfishes (Buckman & Ogden, 1973; Mumby & Wabnitz, 2002). In general, focal TPs interacted agonistically with intraspecific IPs more often than intraspecific TPs, but interactions with intraspecific TPs were longer. Most agonistic interactions between focal TPs and intraspecific IPs appeared to be dominance interactions for access to specific foraging areas within territories and ended quickly with IPs vacating the area. However, some agonistic interactions between intraspecific pairs of TPs and IPs were more aggressive and could have been territorial interactions with IPs belonging to neighboring harems. Agonistic interactions between focal TPs and other intraspecific TPs often involved longer displays or more aggressive chases, indicative of territory defense.

We observed fewer agonistic interactions and greater spatial overlap of home ranges between pairs of interspecific TP parrotfishes. Resource partitioning among species may limit interference competition and enable coexistence among parrotfishes without strong spatial partitioning. Adam et al. (2015) found that nine Caribbean parrotfishes, including *Sc. taeniopterus*, *Sc. vetula*, *Sp. aurofrenatum*, and *Sp. viride*, frequent different habitat types, forage on different substrates (e.g., dead coral and rubble), and target different food types (e.g., turf algae and macroalgae). Recent studies found that parrotfishes partition trophic resources even more finely, targeting substrates in different taphonomic stages characterized by distinct microautotrophic communities (Nicholson & Clements, 2020, 2021). Alternatively, it is possible that interspecific parrotfishes avoid one another dynamically within shared areas of their home ranges to limit interference competition for resources. Future studies of parrotfish movement and space use in shared areas will elucidate the importance of spatial partitioning in facilitating the coexistence of these fishes.

We have shown that parrotfish territories are remarkably stable over short time frames. Other studies have reported fixed space use on the order of months to years in reef fishes, including *Sp. viride* (Reese, 1973; van Rooij et al., 1996). Additionally, van Rooij et al. (1996) reported that the territory borders of *Sp. viride* remained fixed even after territory ownership changed. We observed a similar pattern following an occupancy change in a TP *Sc. vetula* territory. Fixed patterns of space use within parrotfish territories could have important implications for their top‐down effects on benthic coral reef communities. Parrotfishes are nominal herbivores whose grazing has been positively correlated with coral larval settlement and recruitment (Mumby et al., 2007). Spatial constraints on grazing could generate a more suitable habitat for coral larval settlement and recruitment, and affect reef recovery following disturbance (Eynaud et al., 2016; Sandin & McNamara, 2012).

The effects of individual space use by parrotfishes on patterns of benthic community assembly remain poorly studied on coral reefs, though there is some evidence that parrotfishes concentrate foraging within core areas of their home ranges (Carlson et al., 2017; Welsh & Bellwood, 2012). In this study, core use areas represented ~27% of the total home range area of each fish. Constrained grazing by macroalgal browsers (e.g., *Sp. aurofrenatum*; Adam et al., 2015; Dell et al., 2020) within these small areas may drive variation in the local abundances of fleshy macroalgae. Excavating parrotfishes (e.g., *Sp. viride*) remove more of the reef substrate while grazing than scraping species (e.g., *Sc. vetula*), and contribute more to reef bioerosion (Adam et al., 2018; Bellwood & Choat, 1990; Bruggemann, Kuyper, & Breeman, 1994). Additionally, the larger grazing scars created by excavators remain free of algae for longer periods of time (Bonaldo & Bellwood, 2009). As such, constrained grazing by excavators could more effectively maintain bare space for coral larval settlement and recruitment, while also contributing to spatial variation in bioerosion rates. To fully understand the functional roles of parrotfishes on coral reefs, we need a better grasp on species‐specific differences in space use and its effect on foraging patterns.

It is important to note that while we suggest that territoriality is driving the exclusivity and spatial stability of diurnal home ranges in parrotfishes, other processes could generate similar patterns of space use. For example, memory‐based foraging can lead to the emergence of home ranges with limited spatial overlap and improve foraging efficiencies (Benhamou, 1994; Riotte‐Lambert et al., 2015; Van Moorter et al., 2009). We frequently observed focal fishes returning to specific foraging locations within their territories, often in predictable sequences. It is possible that memory‐based movement helps these fishes to forage and defend their territories more efficiently, contributing to the observed patterns of space use.

Studies of animal movement and space use have the potential to advance our understanding of community ecology by connecting patterns to process across multiple spatiotemporal scales (Nathan et al., 2008; Schlägel et al., 2020). Our findings make key advances in our understanding of parrotfish space use on coral reefs and have important implications for the top‐down effects of parrotfishes on benthic coral reef communities. As climate change continues to alter the structure and function of coral reefs, studies of reef fish movement and space use will be increasingly important for understanding how climate change is affecting both reef fish and benthic communities (Manning, 2022).

## AUTHOR CONTRIBUTIONS

**Joshua C Manning:** Conceptualization (lead); data curation (lead); formal analysis (lead); funding acquisition (supporting); investigation (lead); methodology (equal); visualization (lead); writing – original draft (lead); writing – review and editing (equal). **Sophie J. McCoy:** Conceptualization (supporting); funding acquisition (lead); methodology (equal); writing – review and editing (equal).

## CONFLICT OF INTEREST STATEMENT

The authors have no conflicts of interest to declare.

## Data Availability

Data will be made publicly available on Dryad upon acceptance.

## APPENDIX A

**TABLE A1:** Mean (±SE) standard length (cm) and body mass (g) for the initial tracks of the five study species in Summer 2019: *Sc. iseri*, *Sc. taeniopterus*, *Sc. vetula*, *Sp. aurofrenatum*, and *Sp. viride*.

Season	Species	Phase	*N*	St. length (cm) mean ± SE	Mass (g) mean ± SE
Summer	*Sc. iseri*	TP	11	19.64 ± 0.39	81.30 ± 4.59
	*Sc. taeniopterus*	IP	11	16.36 ± 0.51	110.15 ± 7.90
		TP	41	20.15 ± 0.20	179.01 ± 4.12
	*Sc. vetula*	IP	11	22.36 ± 0.72	179.36 ± 15.35
		TP	35	31.40 ± 0.31	474.21 ± 13.04
	*Sp. aurofrenatum*	TP	13	16.46 ± 0.31	67.74 ± 4.38
	*Sp. viride*	IP	12	22.67 ± 0.62	185.65 ± 14.89
		TP	28	29.07 ± 0.38	379.99 ± 13.59

*Note*: We did not record standard length for individuals tracked during Winter 2019 and cannot report mean length and mass.

**TABLE A2:** A full breakdown of GPS tracking replication (*N*) for all seasons, sites, species, and ontogenetic phases; and whether tracks were (✓ or number, when only a subset) or were not (–) used in the statistical analyses of home range (HR) size (full five species model or reduced three species model) and home range overlap (spatial and temporal).

Initial					HR size		HR overlap	
Season	Site	Species	Phase	*N*	5 Species	3 Species	Spatial	Temporal
Summer	AC	*Sc. taeniopterus*	TP	8	✓	✓	✓	–
		*Sc. vetula*	TP	5	✓	✓	✓	–
		*Sp. viride*	TP	4	✓	✓	✓	2
	AQ	*Sc. iseri*	TP	5	✓	–	✓	–
		*Sc. taeniopterus*	IP	5	✓	✓	–	–
		*Sc. taeniopterus*	TP	12	✓	✓	✓	8
		*Sc. vetula*	IP	6	✓	✓	–	–
		*Sc. vetula*	TP	10	✓	✓	✓	4
		*Sp. aurofrenatum*	TP	5	✓	–	✓	–
		*Sp. viride*	IP	6	✓	✓	–	–
		*Sp. viride*	TP	7	✓	✓	✓	5
	BB	*Sc. taeniopterus*	TP	4	✓	✓	✓	–
		*Sc. vetula*	TP	6	✓	✓	✓	–
		*Sp. viride*	TP	6	✓	✓	✓	2
	IV	*Sc. iseri*	TP	6	✓	–	✓	–
		*Sc. taeniopterus*	IP	6	✓	✓	–	–
		*Sc. taeniopterus*	TP	11	✓	✓	✓	6
		*Sc. vetula*	IP	5	✓	✓	–	–
		*Sc. vetula*	TP	9	✓	✓	✓	7
		*Sp. aurofrenatum*	TP	8	✓	–	✓	–
		*Sp. viride*	IP	6	✓	✓	–	–
		*Sp. viride*	TP	6	✓	✓	✓	✓
	TL	*Sc. taeniopterus*	TP	6	✓	✓	✓	–
		*Sc. vetula*	TP	5	✓	✓	✓	–
		*Sp. viride*	TP	5	✓	✓	✓	–
Winter	AC	*Sp. viride*	TP	7	–	–	–	–
	BB	*Sp. viride*	TP	6	–	–	–	–

Replicate					HR size		HR overlap	
Season	Site	Species	Phase	*N*	5 Species	3 Species	Spatial	Temporal
Summer	AQ	*Sc. taeniopterus*	TP	8	–	–	–	✓
		*Sc. vetula*	TP	4	–	–	–	✓
		*Sp. viride*	TP	5	–	–	–	✓
	IV	*Sc. taeniopterus*	TP	6	–	–	–	✓
		*Sc. vetula*	TP	7	–	–	–	✓
		*Sp. viride*	TP	6	–	–	–	✓
Winter	AC	*Sp. viride*	TP	2	–	–	–	✓
	BB	*Sp. viride*	TP	2	–	–	–	✓

**TABLE A3:** The mean (±SE) home range (HR) and core use (CU) areas (m^2^) calculated using movement‐based kernel density estimation (MKDE), location‐based kernel density estimation with ad hoc determination of the smoothing parameter (HOC), and minimum convex polygons (MCP) for initial and replicate GPS tracks of each species and phase during Summer and Winter 2019.

Initial	Species	Phase	*N*	MKDE HR	MKDE CU	HOC HR	MCP HR
				Mean ± SE	Mean ± SE	Mean ± SE	Mean ± SE
Summer	*Sc. iseri*	TP	11	249.15 ± 21.01	65.90 ± 7.85	509.53 ± 123.07	195.96 ± 28.85
	*Sc. taeniopterus*	IP	11	131.42 ± 9.83	35.70 ± 3.21	169.43 ± 18.11	73.82 ± 7.21
		TP	41	122.89 ± 6.78	31.08 ± 1.87	158.78 ± 16.78	65.07 ± 5.23
	*Sc. vetula*	IP	11	154.92 ± 13.66	39.67 ± 2.80	228.71 ± 32.54	94.85 ± 13.22
		TP	35	243.57 ± 14.77	70.07 ± 4.24	382.59 ± 34.24	185.82 ± 16.03
	*Sp. aurofrenatum*	TP	13	199.21 ± 19.39	51.15 ± 6.13	288.25 ± 39.56	128.07 ± 16.86
	*Sp. viride*	IP	12	187.95 ± 19.06	51.47 ± 6.66	314.17 ± 40.99	133.08 ± 18.93
		TP	28	287.43 ± 17.60	80.96 ± 5.65	491.61 ± 44.39	220.22 ± 16.93
Winter	*Sp. viride*	TP	13	304.86 ± 30.13	87.11 ± 9.33	466.61 ± 57.85	272.38 ± 33.21

Replicate	Species	Phase	*N*	MKDE HR	MKDE CU	HOC HR	MCP HR
				Mean ± SE	Mean ± SE	Mean ± SE	Mean ± SE
Summer	*Sc. taeniopterus*	TP	14	141.24 ± 18.52	34.16 ± 3.89	228.90 ± 75.17	110.77 ± 39.24
	*Sc. vetula*	TP	11	231.97 ± 28.73	67.04 ± 8.13	351.16 ± 52.83	174.78 ± 25.65
	*Sp. viride*	TP	11	296.68 ± 24.11	87.13 ± 8.56	447.12 ± 45.25	226.92 ± 24.95
Winter	*Sp. viride*	TP	4	266.66 ± 45.86	74.80 ± 15.67	406.39 ± 92.64	217.25 ± 39.59

*Note*: *N* is the number of tracks conducted.

**TABLE A4:** Mean (±SE) spatial overlap (Bhattacharyya's Affinity) of MKDE home ranges computed for all pairs of spatially co‐occurring terminal phase parrotfishes with the terminal phase *Sc. vetula* that took over occupancy of one of the initial terminal phase *Sc. vetula* home ranges at AQ.

Interaction	Type	*N*	Spatial overlap
*Sc. vetula – Sc. vetula*	Conspecific	1	0.001
*Sc. vetula – Sc. iseri*	Heterospecific	4	0.27 ± 0.11
*Sc. vetula – Sc. taeniopterus*	Heterospecific	5	0.24 ± 0.08
*Sc. vetula – Sp. aurofrenatum*	Heterospecific	2	0.43 ± 0.24
*Sc. vetula – Sp. viride*	Heterospecific	4	0.17 ± 0.15

**TABLE A5:** The mean (±SE) number of GPS relocations and the mean (±SE) and range of time between each relocation (s) obtained during initial and repeat GPS tracks for each species during Summer and Winter, summarized by phase.

Initial	Species	Phase	*N*	Relocations	Relocation intervals (s)	
				Mean ± SE	Mean ± SE	Range
Summer	*Sc. iseri*	TP	11	71.00 ± 3.76	12.65 ± 0.45	1–23
	*Sc. taeniopterus*	IP	11	50.27 ± 2.71	13.37 ± 0.67	1–23
	*Sc. taeniopterus*	TP	41	52.00 ± 1.69	12.93 ± 0.38	1–24
	*Sc. vetula*	IP	11	53.91 ± 2.85	12.86 ± 0.76	1–26
	*Sc. vetula*	TP	35	73.49 ± 2.82	13.41 ± 0.46	1–25
	*Sp. aurofrenatum*	TP	13	56.77 ± 2.17	14.34 ± 0.32	1–23
	*Sp. viride*	IP	12	61.67 ± 7.49	12.36 ± 1.09	1–26
	*Sp. viride*	TP	28	78.82 ± 3.72	12.83 ± 0.52	1–24
Winter^a^	*Sp. viride*	TP	13	175.15 ± 9.53	5.78 ± 0.27	1–14

Repeat	Species	Phase	*N*	Relocations	Relocation Intervals (s)	
				Mean ± SE	Mean ± SE	Range
Summer	*Sc. taeniopterus*	TP	14	58.43 ± 2.61	11.31 ± 0.50	1–22
	*Sc. vetula*	TP	11	80.00 ± 4.32	12.30 ± 0.53	1–54
	*Sp. viride*	TP	11	82.73 ± 3.77	12.47 ± 0.42	1–21
Winter	*Sp. viride*	TP	4	186.00 ± 13.22	5.59 ± 0.38	1–98

*Note*: *N* is the number of tracks conducted.

^a^ This count excludes the TP *Sp. viride* that were tracked in both January 2019 and Summer 2019 at AC and BB (*n* = 2 per site). Those are instead included as repeat tracks here.

**TABLE A6:** The results (Type III Wald's *χ* ^2^) of the best fit and most parsimonious generalized linear mixed model (gamma distribution with a log‐link function) of home range size that included data for all five species and species as the only predictor.

	Wald's *χ* ^2^	Df	*p*‐value
Intercept	6804.60	1	<.001***
Species	34.49	4	<.001***

	Variance	SD
Site	4.85 × 10^−10^	2.20 × 10^−5^

*Note*: The model also included the site as a random effect and a log of track duration as an offset.

^*^ *p* < .05; ^**^ *p* < .01; ^***^ *p* < .001.

**TABLE A7:** The results (Type III Wald's *χ* ^2^) of the best fit generalized linear mixed model (beta distribution with a logit‐link function) of spatial overlap between co‐occurring TP home ranges that included interaction type in both the mean and precision equations of the model (i.e., variable dispersion).

	Wald's *χ* ^2^	Df	*p*‐value
Intercept	755.12	1	<.001***
Interaction type	137.39	14	<.001***

^*^ *p* < .05; ^**^ *p* < .01; ^***^ *p* < .001.

**TABLE A8:** The results (Type III Wald's *χ* ^2^) of the best fit generalized linear mixed model (negative binomial distribution) of the frequency of agonistic interactions between focal parrotfishes (*n* = 114) and other parrotfishes that included focal species, interactor type, and their interaction as predictors.

	Wald's *χ* ^2^	Df	*p*‐value
Intercept	1129.76	1	<.001***
Species	14.44	4	.006**
Interaction Type	92.75	3	<.001***
Species x Interaction Type	30.50	12	.002**

	Variance	SD
Site	9.28 × 10^−10^	3.05 × 10^−5^

*Note*: The model included the site as a random effect and a log of observation time as an offset.

^*^ *p* < .05; ^**^ *p* < .01; ^***^ *p* < .001.

**TABLE A9:** The results (Type III Wald's χ^2^) of the best fit generalized linear mixed model (gamma distribution with log‐link function) of the duration of agonistic interactions between focal parrotfishes (*n* = 114) and other parrotfishes that included focal species and interactor type as predictors.

	Wald's χ^2^	Df	*p*‐value
Intercept	895.53	1	<.001***
Species	12.86	4	.01*
Interaction type	165.08	3	<.001***

	Variance	SD
Focal identity within site	0.08	0.28

*Note*: The model also included focal fish identity within the site as a random effect.

^*^ *p* < .05; ^**^ *p* < .01; ^***^ *p* < .001.
