# Corrigendum

**DOI:** 10.1002/ece3.9877

**Published:** 2023-03-12

In the article by Manning and McCoy (2023), the authors would like to correct the Data Availability Statement as shown below:

All data are available on Dryad at: https://doi.org/10.5061/dryad.h44j0zpms.

The authors apologize for the error.
